# New Dimensions in Patient–Physician Interaction: Values, Autonomy, and Medical Information in the Patient-Centered Clinical Encounter

**DOI:** 10.5041/RMMJ.10085

**Published:** 2012-07-31

**Authors:** Aakash Kumar Agarwal, Beth Brianna Murinson

**Affiliations:** 1 Department of Family Medicine, Kaiser Permanente Los Angeles Medical Center, Los Angeles, California, USA;; 2 Department of Neurology, Rambam Healthcare Campus, Haifa, Israel; and; 3 Department of Neurology, The Johns Hopkins School of Medicine, Baltimore, Maryland, USA

**Keywords:** Clinical, internet, medical education, medical interview, patient-centered care, technology

## Abstract

Patient–physician interactions are increasingly influenced by the extraordinary diversification of populations and rapid expansion of medical knowledge that characterize our modern era. By contrast, the patient–physician interaction models currently used to teach medical trainees have little capacity to address these twin challenges. We developed a new model of patient–physician interaction to explicitly address these problems. Historically, models of patient–physician interaction viewed patient autonomy and the manifestation of clearly defined health care-related values as tightly linked, and it was assumed that patients’ medical knowledge was low. Unfortunately, this does not adequately represent patients such as 1) the highly educated non-medical specialist who possesses little familiarity with health-related values but is highly autonomous, and 2) the patient from a non-Western background who may have well-established health care-related values but a low sense of personal independence. In addition, it is evident to us that the assumption that all patients possess little medical knowledge can create alienation between patient and physician, e.g. the well-informed patient with a rare disease. We propose a paradigm that models autonomy, health care-related values formation, and medical knowledge as varying from patient to patient. Four examples of patient types are described within the context of the model based on clinical experience. We believe that adopting this model will have implications for optimizing patient–physician interactions and teaching about patient-centered care. Further research is needed to identify relevant patient types within this framework and to assess the impact on health care outcomes.

## INTRODUCTION

Over the last few decades, society as a whole has undergone extraordinary shifts that place new strains on the patient–physician relationship. By contrast, the models used for teaching medical students about the patient–physician interaction have remained relatively static.^1^–^4^ Societal expectations, medical sophistication, technological advances, and increased social diversity have all contributed to a new medical world in which patients are more diverse and the availability of medical information is widespread.^5^–^11^ At the same time, increasing pressures for economic efficiency have mandated ever-briefer consultations.^12^ Together, these changes have placed new, perhaps conflicting, expectations on the modern physician.^13^ Unfortunately, the traditional models of patient–physician interaction used for teaching medical students about clinical interactions do not capture the changing face of medicine.

Thus, young physicians are struggling to efficiently incorporate a modern patient dynamic within an old conceptual framework and desperately need a new model of patient–physician interaction that embodies the current realities of medical practice.^14^ This report describes a multidimensional model of patient-centered interaction that addresses the impact of increased patient diversity and medical awareness. Construction of the model is described in two phases: the first phase involves deconstructing previous models of patient–physician interaction in which two variables, namely health-related values and patient autonomy, were tightly coupled in the past.^1^,^15^ The second phase incorporates the possession of medical knowledge by patients as an added new dimension in the patient–physician dynamic.^10^,^16^ This model views patient–physician interaction as varying with the extent of a patient’s formation of health-related values, sense of autonomy, and familiarity with medical information. Several examples illustrating the use of these factors to promote efficient medical practice are presented. We begin by briefly reviewing the evolution of traditional models of patient–physician interaction and establishing necessary definitions.

## TRADITIONAL MODELS OF CLINICAL INTERACTION

Before and during much of the twentieth century, the relationship between physician and patient was typically patriarchal.^2^ Society acknowledged that physicians had exclusive access to medical knowledge and special experience with health-related values and were thus in the best position to make medical decisions on behalf of the patient. Consequently, the physician usually played a dominant role in clinical encounters, and patients abided by physician decisions, while sometimes suppressing their own inclinations. However, with the reshaping of ideals in society, patients became decreasingly satisfied with this stereotypical interaction, and many began seeking greater involvement in the clinical encounter.

Consequently, medical educators developed tools to assist young medical students in understanding the dynamic nature of the patient–physician interaction. What emerged was a series of clinical models that formalize the clinical encounter.^1^ Most widely studied is the four-part classification system described by Emanuel and Emanuel, in which the patient–physician interaction is described as one of four possible types—paternalistic, deliberative, interpretive, or informative—distinguished by the formation of patient values, assignment of decision-making responsibilities (autonomy), and physician disclosure of medical information.

The *paternalistic* scenario describes the “traditional” approach and describes a situation in which the patient has poorly formed values regarding the medical situation. The physician independently decides the interventions to be taken, providing the patient with minimal medical information. Indisputably, there are important medical scenarios where paternalistic care is still necessary, especially in the setting of acute or trauma care where immediate treatment must be rendered and, barring non-resuscitation orders, there is little room for negotiation.

Representing a degree of increased patient involvement is the *deliberative* scenario. The patient in this scenario has minimally formed values, but the physician works with the patient to discover and develop these values. The physician presents carefully selected medical information to the patient. Decision-making is a shared effort, but the physician encourages specific recommendations based on an interpretation of established health-related values.

Continuing in the direction of greater patient involvement is the *interpretive* scenario, in which the patient has inchoate values regarding the situation which the physician helps to elucidate. Substantial dialogue regarding the condition and interventions is exchanged between physician and patient. Once presented with the pertinent information, the patient makes the decision, with the physician acting mainly as a counselor.

Lastly is the *informative* scenario, where patient autonomy is high and the patient has well-formed values; the patient alone takes on decision-making responsibilities. The physician’s role is as a conduit of all relevant medical information.

In the Emanuel and Emanuel system of understanding the patient–physician interaction, the prior formation of patient values, the extent of autonomy, and the amount of medical information *provided to the patient by the physician* are all coupled and change simultaneously. Thus the *paternalistic* model is characterized by low values formation, low autonomy, and low information disclosure, while high values formation, high autonomy, and high information delivery are found in the *informative* model.

In the intervening decades, additional models of patient–physician interaction have examined aspects more or less addressed in the Emanuel and Emanuel model. To this end, Charles and colleagues created a model examining the interplay of patient autonomy and information exchange, stressing that the combination of these and other variables exists on a continuum, rather than at the discrete points suggested by Emanuel and Emanuel.^17^ Bradley and colleagues, recognizing the likely influence of family and friends in decision-making, developed a model where the key players in decision-making served as central variables.^9^ Humphrey et al. developed a model incorporating physician interaction style and patient coping ability, while others have further examined the role of injury severity on interaction, or studied the clinical encounter through a complex interplay of cognitive, emotional, and reflective demands.^18^–^20^

## UNDERSTANDING PATIENT VALUES AND AUTONOMY

Patient values and patient autonomy are central variables in many models of patient–physician interaction. To assist in understanding exactly why this is the case, and to facilitate further discussion, it would be helpful to first consider definitions of these terms.

The term *value* itself is generally defined as the beliefs or principles of a person or group that are used to guide decisions and way of life.^21^ Collectively, values give weight and worth to ideas and actions. A person’s values strongly influence how one feels about many issues, including choice of occupation, the utility of preserving life, and expenditure of resources on various items. The formation of these values is an important developmental task of young adults, but an individual’s awareness of these values continues to develop over the course of a lifetime, a product of upbringing, interaction with others, and a variety of life experiences. *Health-related values* specifically describe a person’s values relating to the medical sphere, and the impact of these values on treatment choice and commitment to health-sustaining activities. Health-related values include the extent to which a person values life versus lifestyle, personal health versus preservation of family assets, and unpleasant physical symptoms versus potential health benefits.

*Patient autonomy* concerns the patient’s right to involvement in the discussion and decision-making process during consultation.^3^ It can further be described as the patient’s ability to make medical care decisions without being influenced too strongly by care providers or others. Respect for patient autonomy is an important tenet of ethical medical conduct and reflects a balance of the physician’s practice style with the patient’s inclinations. A common challenge to patient autonomy arises when the patient’s expressed preferences contradict what the physician perceives as being in the patient’s best interest, such as when the patient refuses necessary treatment or expresses desires drastically different from those of family and friends.^22^,^23^ Patient autonomy falls on a wide spectrum, ranging from very high, where patients make all decisions, to very low, where they have minimal decision-making involvement. Patient autonomy is often associated with the idea of “locus of control,” which emerged from Julian Rotter’s Social Learning Theory, where personality is described as the product of individual and environment.^24^ Locus of control describes the extent to which one feels in control of one’s environment and has been explicitly extended to health care through such tools as the Multidimensional Health Locus of Control Scales (MHLC).^25^ The MHLC describes a person’s sense of control as “internal” if the person views their health outcome as in their hands, as “external/chance” if health outcome is viewed as the result of outside luck or chance, or as “external/powerful” if it is the product of a strong outside entity, including health care providers. The concept of health-related locus of control has been studied carefully with respect to areas such as palliative care and sports medicine among others, with higher internal control being commonly associated with overall improved health outcomes.^22^,^26^–^28^

## FORMING THE FOUNDATIONS OF A NEW MODEL: BREAKING OLD LINKS

**Figure 1:**
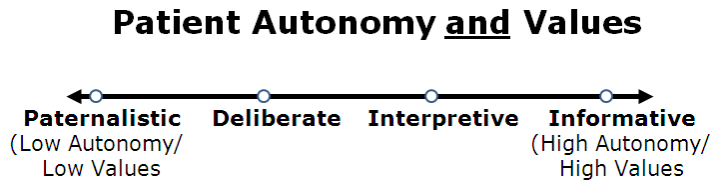
**The Emanuel and Emanuel model.** Patient autonomy and patient values are closely linked and essentially mutually varying. Clinical scenarios described thus fall on a single line.

Because of their strong impact on the nature of patient–physician interaction, patient values and autonomy have been key variables in many past models. However, while most models correctly identify the existence of both variables, they also tightly link these factors and, thus, fail to understand the potential independent expression of values and autonomy in individual patients. For instance, Emanuel and Emanuel imply that as patient autonomy and decision-making involvement increase, the strength and formation of patient values increase as well. This is clear when examining the specifics of their model, where a shift from completely unformed to fully formed values—and a corresponding shift from low patient autonomy to high patient autonomy—occurs as one progresses from the *paternalistic* approach to an *informative* one. Visually, this can be represented as a single axis in which the extent of values formation and patient autonomy are mutually varying (Figure 1).

**Figure 2:**
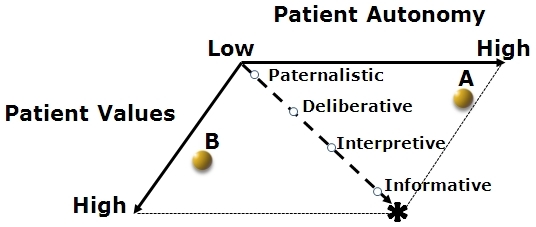
**A reinterpretation of past models.** In past models patient values and patient autonomy have often been tightly linked. These models assume that as values formation increases, autonomy must as well when in fact these variables may not always co-vary. As described in the text, many patients fall away from this diagonal line. Examples of this include **A**, the patient with high levels of autonomy and relatively unformed health care-related values, e.g. a financial analyst, and **B**, the patient from a very traditional culture where health care-related values are clear but personal autonomy is low.

In clinical practice, however, it has become evident that many patients are not well represented by this single-axis approach, e.g. the patient with high autonomy but low formation of health-related values. Consequently, the first step in the formation of the new model is to allow autonomy and health care-related values to vary independently of one another. This can be represented by plotting values and autonomy on separate, perpendicular axes as illustrated in Figure 2, which expands the single axis (spectrum) of previous models into a two-dimensional space.

An example of a situation in which values and autonomy are uncoupled could be a stock analyst or high-ranking business executive recently diagnosed with a rare disorder. From years of experience with executive responsibilities, this patient has a high decision-making capacity and may have a seemingly compulsively desire to be deeply involved with all decisions and actions taken. Coming from outside of the medical sphere, however, this patient may have no familiarity with the nature of illness or with health care as a whole. This patient may be completely out of sync with translating general values into health-related decisions and may have given little forethought to the advantages and disadvantages of various diagnostic procedures and treatment alternatives. This is a patient whose level of autonomy is high, while the extent of values formation, especially as it relates to health care, is low. This patient (**A** in Figure 2) falls outside of the categories found in traditional models and requires modified approaches to ensure a meaningful and successful patient–physician interaction. A physician relying on traditional models may mistakenly assume a linkage of values formation and autonomy. As a result, the physician might conclude that the patient has strong formation of health-related values to complement the high level of autonomy. Alternatively, the physician might think that the patient desires low autonomy because of the low prior formation of health-related values. Both of these mistakes on the part of the physician will result in a less than ideal clinical encounter. The failure to recognize that enhanced support regarding health care-related values would be helpful to this patient may 1) lead the patient to choose a path of excessive medical intervention with attendant risks and costs, or 2) lead the patient to mistakenly avoid appropriate assessment and intervention. Conversely, by neglecting the patient’s inclination to high autonomy, the physician risks alienating the patient, appearing oblivious to the need for independence in decision-making. One solution, suggested by the new model, is to recognize that this patient will need at least part of the clinical encounter to focus on exploring and developing health-related values. The physician must be aware that once the appropriate values are elucidated, it will be necessary to proceed in a way that respects the need of this patient to be highly autonomous in decision-making.

An alternative example of the independence of patient values and autonomy is the patient whose culture of origin emphasizes the primacy of the family unit in decision-making and places less value on autonomy. Each decision, regardless of its implications, can only be undertaken following intensive interaction with family and friends; in many cases, the patient will not make even minor decisions alone. This patient may actually have strong values regarding his or her condition, but this patient lives within the framework of shared values of the extended family. This is a patient whose level of autonomy is low, while the extent of values formation may be high (**B** in Figure 2. These patients can be especially confounding for physicians familiar only with the traditional models in which the patient with clearly established values is highly autonomous. Failure to recognize this pattern can result in the physician negotiating treatment with the patient, which subsequently fails to be implemented. This may lead the physician to wrongly conclude that the patient is wayward or non-compliant, when in fact a distinct dynamic is at work. In some cases, life-saving interventions are postponed, or unnecessary suffering occurs, due to the resulting delays in communications and decision-making. In an ethnically diverse society comprised of different cultures that embrace specific ideals and varying decision-making styles, physicians must be prepared to recognize, validate, and work with these differences in the decision-making process. In looking to accommodate this patient’s approach, the physician should seek to include family members in the clinical encounter. For reasons of intimacy and efficiency, the physician must further encourage the physical presence of contributing family members by integrating them into the process.

As the above two examples illustrate, there are common clinical scenarios which will fall significantly away from the reduced axis implied by so many past models in which values and autonomy mutually vary. Hence, it is necessary to create two separate axes in order to emphasize the independence of values and autonomy. In this way, the range of clinical scenarios is more realistically represented, expanding from a single line to an entire plane of possibilities. This creates a framework for anticipating the broader range of possibilities inherent to modern, diverse patient populations.

## CONTINUING CHANGE: THE INFORMATION REVOLUTION

In addition to increased patient diversity, the last several years have seen a profound increase in medical information available to the public. Whether simply the result of emerging avenues of communication, or the aftermath of consumer criticism of medical community monopolization of scientific knowledge, there has been an undeniable increase in the publication of medically relevant texts, journals, magazines, and direct-to-consumer advertising in print and electronic media. World-wide access to information through the internet has been the most important factor in this exponential growth of medical knowledge accessibility. As we enter what some have dubbed the “Internet Age,” more people have immediate access to medical information. It is estimated that billions of people world-wide use the internet. In North America, the internet was available in 70% of homes in 2009, the latest year for which statistics are available.^29^ Additionally, the value of the internet as a source of information is unlike that of any other existing tool. A multitude of websites are designed for people of all ages, education levels, and general background demographics, allowing many individuals to turn to the web to research medical questions.

As a consequence of the growing availability of information accessible to the general public, a new dynamic within clinical interaction has emerged, greatly impacting the medical sphere and how patients view their condition. Studies have found that a significant percentage of American patients, ranging from close to 30% to over 50%, have used the internet as a resource for medical information,^10^,^16^,^30^ and that more than 100 million adults have surfed the web in search of health and medically related matters.^31^ For patients, having additional knowledge has often been reported as overwhelmingly helpful, as it gives them more confidence to speak with their physician (97%), encourages them to follow their doctor’s advice (85%), enables them to understand their problem better (86%), benefits them in the decision-making process (74%), and improves their communication with their doctor (62%).^10^

But for many health care providers, this new source of information induces an unfamiliar dynamic. While it is estimated that the majority of physicians utilize the internet themselves,^10^ an astonishingly low percentage discuss the internet as a tool with their patients. Most commonly, physicians have expressed concern over the validity of the information found on the internet, especially in the hands of untrained patients. One study found that 87% of surveyed physicians were concerned about the quality of medical information available to their patients, and 84% expressed further concern over their patients’ ability to judge information quality adequately.^10^ A number of recent studies have further examined the reliability of such medical information and found less than desirable results. Culver et al., for instance, examined an online discussion group and found that 90% of the medical advice presented was offered by contributors with no medical background.^32^ A study by Impicciatore et al. found that less than 10% of patient-oriented health websites adhered closely to published guidelines, with some even suggesting potentially harmful therapies.^33^ Consequently, physicians remain wary of these tools and seem additionally concerned over the possible impact that inaccurate and inappropriate internet information may have on their patients and their interaction, believing such information gives rise to false hope, anxiety, and knowledge.^6^

Patients and physicians thus remain somewhat at odds over how to incorporate patient-researched medical information into the clinical encounter. Though physicians have largely shown discomfort with patients utilizing outside information as tools, patients continue to express a strong desire for greater physician involvement in their own searches for information.^5^,^34^ Consequently, there is a pressing need to address this issue in an effort to assist physicians in preparing for this new dimension in modern medicine.

## THE ADDED DIMENSION IN PATIENT–PHYSICIAN INTERACTION: PATIENT MEDICAL KNOWLEDGE

**Figure 3:**
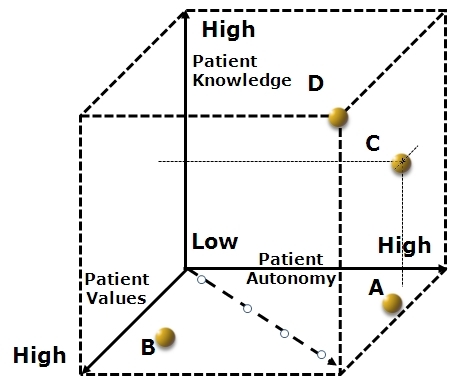
**Our model.** Patient values, patient autonomy, and patient knowledge are the three axes in our model, emphasizing both their independence and interaction. Included also is the “Emanuel and Emanuel Reduced Axis,” which implies a mutual variability with patient autonomy and values, and plotted examples (**A, B, C, D**) highlighting the necessity of stepping away from the simplifications implied by past models. See text for details. **A and B:** The same as in Figure 2; **C**: patients may be selectively well-informed about specific diseases; **D:** Highly informed patient such as a physician.

In previous models, the impact of patient medical knowledge was not formally incorporated. The flow of medical information was assumed to move only from physician to patient, but with information becoming increasingly available to patients, such an assumption is no longer reasonable. Patient familiarity with technical material has begun to significantly influence the dynamic of patient–physician interactions. As such, we complete our model of patient–physician interaction with the addition of patient medical knowledge as a third and final axis. Patient medical knowledge thus joins patient values and patient autonomy as the central variables considered in our discussion. The essence of our model design is shown in Figure 3. Two additional examples of patients, for whom medical information has a substantial impact on care, will be discussed.

The first is a patient who arrives with a significant degree of medical knowledge after being diagnosed with a rare disorder and actively pursuing information pertaining to their syndrome, the “informed patient” (**C** in Figure 3). In reality, the informed patient is becoming much more common and represents a significant challenge to the traditional modes of communication between patient and physician. Often highly autonomous, these patients may have a very clear sense of what an ideal patient–physician interaction is like for them. Recognizing that not all physicians will be intimately familiar with each rare condition, the informed patient may come to view themselves as an “expert consultant on syndrome X.” For their part, the physician faces the challenge of gauging the extent and accuracy of this patient’s medical knowledge and adapting the clinical encounter to the patient’s needs. If the physician, operating under the traditional models, refuses to acknowledge the medical information that this patient has acquired, both patient and physician will be frustrated in the encounter. Our model suggests some ways in which the clinical encounter can adapt to this new challenge. The first step is to assess the degree of autonomy, values, and information that that patient possesses. As indicated by the location of point “C” in Figure 3, the example patient has high autonomy, modest values formation, and moderate medical knowledge. Thus, this patient will benefit from guidance in forming appropriate health-related values, which will be an important part of the clinical encounter. Additionally, the informed layperson will not have the benefit of a comprehensive medical education and will still need general medical care and counseling in the context of a rare condition, for example, the management of high blood pressure (a common condition) in a patient with Stiff-person syndrome (a rare condition). The physician can provide guidance about the use of specific websites that convey well-vetted and reliable information. Thus by assessing the patient for levels of autonomy, values, and medical knowledge, the physician can more accurately calibrate their contributions to the interaction to better meet the needs of the patient.

An example of someone entering the medical encounter with an extreme degree of medical knowledge is the physician-as-patient (**D** in Figure 3). In the case of a physician seeking medical care, the discussion of medical information is often brief, revolving around clarifying some points of detail or highlighting the very latest developments within a field. As a rule, the physician-as-patient expects to exercise a high degree of autonomy, and this can be quickly confirmed by the treating physician. What may be less certain is the capacity of the physician-as-patient to identify and apply their professionally held health-related values to their own medical condition. Sometimes it is especially difficult for a physician to shift into the role of patient. A focused effort on the part of the treating physician to acknowledge this difficulty and explore the extent to which health-related values are being properly applied can reduce feelings of isolation and distress. Minimizing patient distress is always important for genuine patient–physician interaction, because it is often only when a patient feels truly comfortable that the most critical concerns come to the surface.

The addition of the medical information axis in our model highlights that patients may now arrive to their appointments equipped with substantial medical knowledge, where such instances were rare decades ago. The traditional models of patient–physician interaction describe the exchange of patient information as a contributing factor, but always imply a unidirectional flow of medical knowledge from physician to patient. Consistent with this, physicians of the past held the power to control exclusively the flow of medical information, and thus uniquely dictated the course of discussion. This meant that physicians needed those communication skills that facilitated the clear explanation of medical facts and interventions to patients of varying backgrounds and education levels. As patients have become increasingly knowledgeable, the flow of medical information has become bidirectional, and now patients are often able to engage in meaningful knowledge-based dialogue. For most physicians practicing today, this represents a significant change in the clinical dynamic that will require the cultivation of new communication skills, as discussed below. Nonetheless, our model proposes the idea that by assessing patient autonomy, values, and medical knowledge, the patient–physician interaction will be enriched.

## DISCUSSION

Our proposed model emphasizes the critical interplay of traditionally recognized variables, specifically the formation of patient values and patient autonomy together with the increasingly important element of patient medical knowledge. While past models may have once represented the essential features of the patient–physician interaction, recent societal and medical changes have impacted clinical medicine such that a new model is needed to portray modern populations accurately. Undue reliance on an oversimplified model promotes the infringement of patient care, as physicians struggle to accommodate new patient dynamics into their existing and inadequate schemas. With the introduction of added variables, however, physicians will be better prepared to appreciate fully the nature of their patients and generate ideal approaches for each.

This multidimensional model of patient–physician interaction importantly highlights the growing influence that patient medical knowledge will have on clinical encounters and encourages physicians to address these changes effectively for the benefit of their patients. In part due to the vast resources poured into biomedical research, there has been an explosion of detailed medical information available regarding any number of medical conditions. Indeed, one of the major concerns for medical faculty involved in medical education has been the question: “What do we teach student doctors when they can no longer know everything about medicine?” While 50 years ago it may have been reasonable to expect a comprehensive knowledge of the wider scope of medicine, advances in genetics, molecular biology, medical technology, and other aspects of medicine have made this an impossible goal.^35^ Some approaches include focusing on teaching about the more common conditions, or conditions that would have catastrophic results if undiagnosed, or focusing on conditions that especially clarify a particular pathway or mechanism. Many educators have also adopted the approach of promoting lifelong learning skills. Yet, often neglected is the impact of these changes on the patient–physician interaction. In this new medical paradigm, the physician is often not the sole repository of medical information, which means that the patient–physician interaction is negotiated anew each time a knowledgeable patient is encountered, an unacceptably inefficient approach. However, the new model of patient–physician interaction will facilitate the development of new communication strategies, especially those focusing on providing patients with a broader context of medical knowledge, guiding patients to reputable sources of information, and promoting the development of health-related values.

A necessary future step in the further development of our new paradigm of patient–physician interaction includes a careful study of patient populations within the context of this model framework (see Table 1). This would afford better understanding of the most commonly encountered patient archetypes and would further highlight those having the greatest impact on clinical outcomes. We have described four patient types that may be encountered in clinical practice and that serve to illustrate the pressing need for this new approach. By surveying patient populations with respect to autonomy, values, and medical knowledge, it will be possible to identify which patient types are most often seen. This will allow physicians to recognize patient types more quickly and understand more clearly which clinical approaches are most needed. Moreover, such research may allow the identification of important patient classifications that have so far been unidentified.

**Table 1:** Framework for classification of patients in terms of degree of autonomy, formation of health care-related values, and extent of medical information.

Low Medical Information*		**High Autonomy**	**Moderate Autonomy**	**Low Autonomy**	**No Autonomy**
	Very well-formed health care values	Informative		Persons from groups with characteristically low autonomy	
	Moderately well-formed health care values		Interpretive		
	Few well-formed health care values	Technical specialist, e.g. financial analyst		Deliberative	
	No well-formed health care values				Paternalistic, trauma care
**Moderate Medical Information**		**High Autonomy**	**Moderate Autonomy**	**Low Autonomy**	**No Autonomy**
	Very well-formed health care values				
	Moderately well-formed health care values	Well-informed patient with rare disease			
	Few well-formed health care values				
	No well-formed health care values				
**High Level of Medical Information**		**High Autonomy**	**Moderate Autonomy**	**Low Autonomy**	**No Autonomy**
	Very well-formed health care values	Health professional as patient			
	Moderately well-formed health care values				
	Few well-formed health care values				
	No well-formed health care values				

* On the part of the patient, traditional models

Collectively, the theoretical and research work in regard to new patient–physician models for clinical interaction will better prepare both experienced and newer physicians for the modern patient population. Especially with regard to student doctors, as the foundations for their future practice are actively forming, limiting study to older models could adversely impact their understanding of real-life patient encounters. Exploration of our new model, in contrast, will allow young physicians to consider early on how newer variables impact the clinical dynamic, and thus adjust their approach accordingly.

The importance of accurate models of patient–physician interaction cannot be overstated as physicians who seem unable or troubled in adjusting to the modern dynamic have been associated with poorer care. In studying this issue, Murray et al. found that a physician’s feeling of being challenged was a significant predictor of perceived deterioration in the patient–physician relationship. This perceived threat was additionally the leading indicator of worsened quality of care and health outcome. Furthermore, patients displeased with their physician, for reasons pertaining to physician communication skills, reactions to patient information, and appearance of feeling threatened or overly challenged, are often led to seek a second opinion, to change physicians, or even to change health plans entirely.^8^,^10^,^36^ Clearly, a physician’s comfort with the changing dynamic within clinical interaction plays an undeniable role in influencing patient interaction; those who resist conforming to this new variable risk not only serious damage to the patient–physician relationship, but also threaten patient health care.

The need for physicians to acknowledge and understand the increasing impact the internet and other health sources will have on the patient–physician interaction will only continue to grow. Studies have shown that while patients do indeed have the greatest trust for physicians, younger generations—those less bound to tradition—invest increasing faith in the internet and decreasing reliance on physicians when compared to older patients.^16^ Considering future implications, physicians must learn to integrate the presence of the internet into their own practice. Our model serves as an excellent template for physicians to begin this process and make themselves aware of necessary changes. As both patients and physicians manifest adaptive strategies to better navigate the ever-changing nature of modern medicine in the context of a diverse society, the patient–physician interaction will enter a new, richer phase of development.

## Abbreviations:

MHLC: Multidimensional Health Locus of Control Scales
